# A Cross-Sectional Survey of Healthcare Professionals’ Knowledge, Attitude and Current Behaviours towards Female Fertility Preservation Services within the UK

**DOI:** 10.3390/cancers16152649

**Published:** 2024-07-25

**Authors:** Lorraine S. Kasaven, Anita Mitra, Mehar Chawla, Sughashini Murugesu, Nicholas Anson, Jara Ben Nagi, Efstathios Theodorou, Michael P. Rimmer, Bassel Al-Wattar, Joseph Yazbek, Benjamin P. Jones, Srdjan Saso

**Affiliations:** 1 West London Gynaecological Cancer Centre, Du Cane Road, London W12 0NN, UK; anita.mitra@nhs.net (A.M.); mehar.chawla@nhs.net (M.C.); sughashini.murugesu@nhs.net (S.M.); nicholas.anson@nhs.net (N.A.); joseph.yazbek@nhs.net (J.Y.); benjamin.jones@nhs.net (B.P.J.); srdjan.saso@nhs.net (S.S.); 2 Department of Surgery and Cancer, Imperial College London, South Kensington, London SW7 2AZ, UK; 3 Department of Cutrale Perioperative and Ageing Group, Imperial College London, London W12 0NN, UK; 4 Centre for Reproductive and Genetic Health, Great Portland Street, London W1W 5QS, UK; jara.bennagi@crgh.co.uk (J.B.N.);; 5 Centre for Reproductive Health, Institute for Regeneration and Repair, University of Edinburgh, Edinburgh EH16 4UU, UK; michael.rimmer@ed.ac.uk; 6 Beginnings Assisted Conception Unit, Epsom and St Helier University Hospitals NHS Trust, Sutton SM5 1AA, UK; b.wattar@nhs.net; 7 Clinical Trials Unit, Anglia Ruskin University, Chelmsford CM1 1SQ, UK

**Keywords:** fertility preservation, oncofertility, cross-sectional survey, healthcare professional, cancer

## Abstract

**Simple Summary:**

Healthcare professionals recognise the importance of fertility preservation and their responsibility to initiate discussions. However, despite advanced training within respective fields, discrepancies in knowledge remain regarding techniques of fertility preservation, referral pathways and existing educational resources available to patients, preventing efficient implementation of services. Further education and training regarding the methods of fertility preservation and how to refer patients to specialist services are required. Many of the personal barriers faced by healthcare professionals could be overcome through regular teaching on communication skills used for breaking bad news.

**Abstract:**

(1) Background: This study aims to establish the knowledge, attitudes and current behaviours towards female fertility preservation (FP) services amongst healthcare professionals (HCPs) in the UK. (2) Methods: An online survey was advertised publicly on the social media platform *Instagram* between 25 February 2021 and 11 March 2021. (3) Results: In total, 415 participants fulfilled the inclusion criteria and completed the survey. The majority of HCPs discussed FP techniques either never 39.5% (n = 164), once a year 20.7% (n = 86) or once a month 17.8% (n = 74). The majority rated their knowledge of each type of FP method as ‘very poor’ or ‘poor’ and strongly disagreed 14.2% (n = 59) or disagreed 42.2% (n = 175) with the statement they ‘felt confident to counsel a patient on FP’. The majority either agreed 37.8% (n = 157) or strongly agreed 22.2% (n = 92) that it was their responsibility to discuss FP and 38.1% (n = 158) agreed or strongly agreed 19.5% (n = 81) they considered the desire for future fertility when planning treatment. The majority 87.2% (n = 362) had not experienced formal training on FP. (4) Conclusions: Discrepancies in knowledge remain regarding techniques of FP, referral pathways, awareness of facilities offering services and existing educational resources. Many HCPs recognise the importance of FP and their responsibility to initiate discussions. The knowledge that FP may not delay the treatment of cancer has also improved; however, training in FP is scarce.

## 1. Introduction

One of the biggest healthcare challenges faced in the 21st century is the treatment of cancer. With diagnostic advancements and therapeutic treatments improving significantly in the last few decades, the 5-year survival of cancers in children, adolescents and young adults is more than 80% [1], with some specialities reportedly seeing malignancy cure rates of more than 90% [2]. With the age-standardised mortality rates projected to continue falling by 14.6% amongst females and 18.3% in males between 2014 and 2034 [3], more survivors are expected to live with long-term treatment effects. Given that cancer therapies predominantly include systemic chemotherapy and radiation, which are gonadotoxic in nature, cancer treatment often results in irreversible effects on reproductive potential, including diminished ovarian reserve, premature onset of menopause and ovarian failure in women [4,5]. Certain hormone-receptive cancers requiring prolonged duration of endocrine treatment may also delay the ability to conceive, which in turn increases the risk of age-related fertility decline [6]. Furthermore, the treatment of certain gynaecological cancers may require partial or complete surgical removal of reproductive organs, rendering women infertile. The inability to conceive is a significant quality of life matter for both males and females [7], and is now understood to influence self-esteem and identity [8], cause significant interruption to planning for the future and increase pressure placed on social relationships [9]. 

There are a number of fertility preservation (FP) techniques patients can consider once diagnosed with cancer to preserve their fertility. For males, many adults may opt for sperm cryopreservation, or in cases of ejaculation failure, may undergo epididymal aspiration or testicular biopsy [10]. In pre-pubertal males, testicular tissue may be cryopreserved, although the availability of this to patients is variable and no human has ever been born from cryopreserved testis tissues [11]. The options for females are dependent on various factors including age, diagnosis and type of treatment, whether undergoing FP would delay cancer treatment and relationship status. Common methods of FP include embryo or oocyte cryopreservation. Previously, ovarian tissue cryopreservation was considered an experimental technique; however, given advances within the field, a recent guideline published by the National Institute for Health and Care Excellence (NICE) in 2023 now recommends the removal, preservation and reimplantation of ovarian tissue to restore fertility after gonadotoxic treatment, not just for prepubertal patients, but for adults who also fulfil the criteria [12]. Other methods of FP may include the use of gonadotrophin-releasing hormone (GnRH) agonists or transposition of the ovaries [13]. FP, however, is not without risk as ovarian stimulation may increase the prevalence of cancer in hormone-sensitive malignancies and ovarian or testicular tissue cryopreservation is associated with surgical risks [14].

Concerns regarding fertility can influence patients’ decisions to undergo treatment for cancer [15], which may consequently affect their mortality rate. The role of the healthcare professional (HCP) can be influential in the decision-making process, as women in particular tend to make decisions regarding FP with their HCP [16] and actively seek their support [17]. Evidence suggests the decision-making process is less influenced by non-personalised information provided by national organisations [18], but more by tailored information received from the individual oncology teams. The latter offers stronger support for patients and families and is more likely to encourage them to engage with healthcare services [19]. The role of the HCP, therefore, at initial diagnosis is paramount in potentially influencing decisions regarding cancer treatment and overall mortality, future fertility planning and subsequent quality of life during the survival years. 

The importance of recognising the difficulties surrounding reproductive potential following treatment of cancer is acknowledged, as reflected by many national and international guidelines emphasising the need for HCPs to initiate discussions regarding the implications on future fertility when undergoing treatment of cancer as early as possible, and explain the methods to preserve gonadal function and fecundity with patients of reproductive age [20,21]. In particular, the ASCO Clinical Practice Guideline Update 2018 recommends that HCPs should discuss all FP options and/or refer patients to reproductive specialists, whilst ensuring documentation of such discussions has taken place [21]. However, despite the guidance and advances within assisted reproductive technologies (ART), evidence suggests that female patients receive limited information on fertility risks and only a small number of patients are referred to specialists [22,23]. Furthermore, half or less than half of patients are effectively counselled on FP and a minority undergo treatment [24,25]. The discrepancy between the availability of FP services and its implementation for cancer patients has been attributed to several institutional and personal challenges HCPs face within their day-to-day practice [26,27,28]. Although many qualitative studies have explored the views of HCPs and determined the perceived barriers to implementing FP within a few specialities, the findings reflect the practice of more than ten years ago [27,29,30]. In view of recent advances within the field of FP, further understanding of the knowledge regarding FP amongst HCPs and the institutional barriers they face are required, not only to bridge the gap between oncology and fertility specialists, but to determine how to improve the implementation of FP services to ensure all patients of reproductive age diagnosed with cancer are at least offered, and or, receive FP treatment where clinically indicated. 

The aim of our study is to explore HCP’s current awareness of FP in the UK and identify specific gaps in knowledge or areas within training that may overcome challenges when implementing FP for patients with cancer. 

## 2. Materials and Methods

### 2.1. Study Design and Population

An online survey regarding the knowledge, attitude and current behaviours towards female FP services within the UK was advertised publicly on the social media platform *Instagram*, by a gynaecology UK healthcare professional with over 70,000 followers, between 25 February 2021 and 11 March 2021. The survey was also distributed by representatives of the UK Audit and Research Collaborative in Obstetrics and Gynaecology (UKARCOG) to trainees within their respective deaneries of training, a UK-wide network capable of disseminating and co-ordinating national data collection in Obstetrics and Gynaecology [31]. The survey was aimed at doctors working in either primary or secondary care within the NHS, providing care to women of reproductive age with cancer across all specialities. A hyperlink was shared directing participants to the survey using the platform Qualtrics (Qualtrics.com) with a brief summary and purpose of the study described in the invitation to participate. The summary reiterated that participation was exclusively voluntary with no offer of incentive to complete the survey. The survey consisted of 77 questions designed for quantitative analysis and took approximately 10–15 min to complete. The inclusion criteria included doctors from all specialities who provided care to women aged 18–45 within the UK. Data from participants who did not fulfil the inclusion criteria were excluded from the analysis. 

The survey consisted of seven categories. First participants answered questions regarding demographic characteristics such as age, ethnicity, speciality, level of training, details of undergraduate training, deanery where they completed their training and qualifications (9 questions). This was followed by questions assessing the current practice of FP (12 questions). The Section 3 assessed knowledge of FP (10 questions) and the fourth attitudes towards FP (14 questions). The Section 5 assessed barriers to FP (11 questions), followed by knowledge of FP services (11 questions) and finally questions regarding training of FP (10 questions). The questionnaire is available in Supplementary File S1.

### 2.2. Statistical Analyses

SPSS version 24 software (SPSS Inc., Armonk, NY, USA) was used for the analysis of data. Descriptive statistics included mean ± SD or median ± range. A Shapiro–Wilk test was performed to assess for normality. 

### 2.3. Patient and Public Involvement 

As the survey was trialled on HCPs, respondents were not involved in the study design, its execution or data analysis. Results will be communicated to respondents through the social media platform Instagram (version 318.0), and also via email, to those who requested the findings be shared with them. 

## 3. Results

A total of 433 HCPs completed the survey. However, 18 were excluded from the data analysis due to not fulfilling the inclusion criteria. Therefore, 415 complete surveys were analysed.

The demographics of the respondents are detailed in Table 1. There were 78.6% (n = 326) participants who completed their undergraduate training in the UK, compared to 20.7% (n = 86) who did not and 0.7% (n = 3) who did not answer. The majority also completed their postgraduate speciality training in the UK (90.6%; n = 376), compared to 8.7% (n = 36) who did not and 0.7% (n = 3) who did not answer. The most common cancer pathologies seen in practice are reported in Supplementary Figure S1.

### 3.1. Current Practice of Fertility Preservation

**Figure 1:**
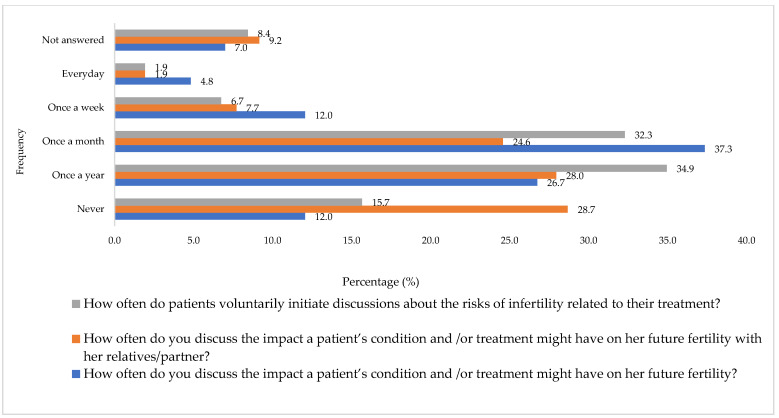
Current practice of fertility preservation.

**Figure 2:**
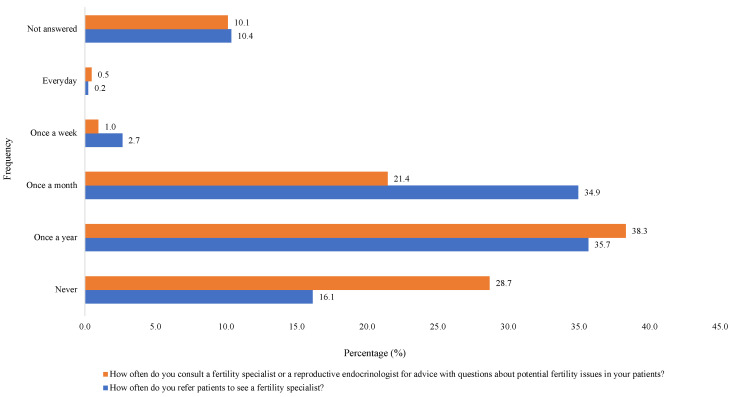
Referral to fertility preservation services.

**Figure 3:**
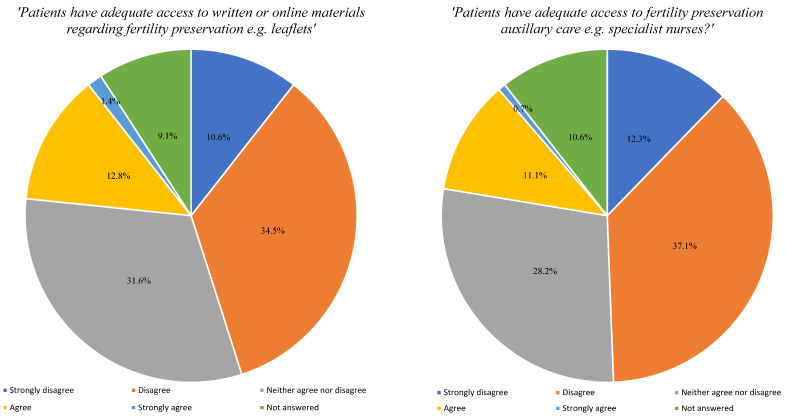
Access to fertility preservation services.

Figure 1 reports the frequency at which HCPs discuss the impact a patient’s condition and or treatment has on future fertility, either with the patient themselves or with parents or relatives and how frequently patients voluntarily initiate such discussions. When asked how frequently HCPs discuss FP techniques during counselling prior to treatment of cancer, 39.5% (n = 164) reported never, 20.7% (n = 86) once a year, 17.8% (n = 74) once a month, 4.6% (n = 19) once a week, 1.7% (n = 7) every day and 15.7% (n = 65) did not answer. The most commonly discussed method of FP was oocyte cryopreservation (29.4%; n = 122), followed by embryo cryopreservation (22.4%; n = 93), ovarian tissue cryopreservation (12.5%; n = 52), GnRH agonist with chemotherapy (12.3%; n = 51), transposition of the ovaries (4.6%; n = 19), experimental techniques such as transplantation of the ovary (0.72%; n = 3) and (18.1%; n = 75) did not answer. Figure 2 reports the frequency HCPs refer patients to FP services. When asked about written resources regarding FP, only 12.8% (n = 53) provided this to patients compared to 60.2% (n = 250) who did not and 26.9% (n = 112) did not answer. Figure 3 reports with a Likert scale how strongly they agree or disagree with patients’ current access to FP resources. Supplementary Figure S2 demonstrates HCP’s satisfaction with the amount of discussion regarding FP with their patients that occurs within their personal practice and within the unit they work in. 

### 3.2. Knowledge of Fertility Preservation

**Figure 4:**
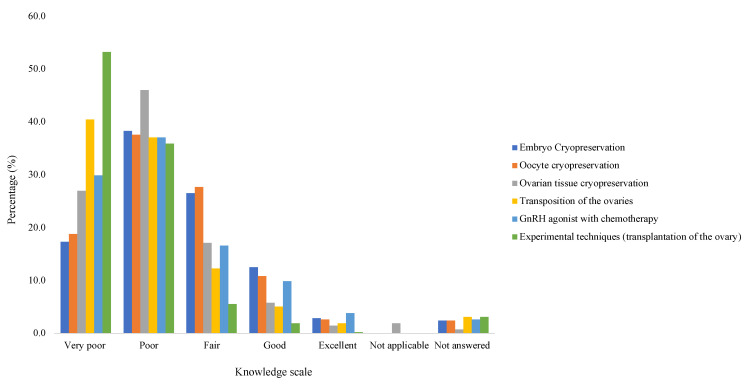
Knowledge of fertility preservation techniques.

HCPs reported they were not at all aware (2.4%; n = 10), slightly aware (15.4%; n = 64), moderately aware (34.9%; n = 145), very aware (24.6%; n = 102) and extremely aware (10.1%; n = 42) of the risks of infertility associated with the treatments (medical or surgical) that they offered to patients of reproductive age. When asked if they felt confident to counsel a patient of reproductive age about FP, 14.2% (n = 59) strongly disagreed, 42.2% (n = 175) disagreed, 15.7% (n = 65) neither agreed nor disagreed, 19.5% (n = 81) agreed, 4.6% (n = 19) strongly agreed and 3.9% (n = 16) did not answer. Figure 4 reports the participant’s knowledge of various FP techniques. A third of the participants (36.4%; n = 151) believed FP would delay treatment of the underlying condition, whereas 19% (n = 79) believed this was not the case, 41.9% (n = 174) were unsure and 2.7% (n = 11) did not answer. Over half the participants (53.3%; n = 221) were aware of the potential health risks to the mother associated with pregnancy after completing treatment for cancer, whereas 17.3% (n = 72) did not think this was the case, 27.5% (n = 114) were unsure and 1.92% (n = 8) did not answer. 

### 3.3. Attitudes towards Fertility Preservation

**Figure 5:**
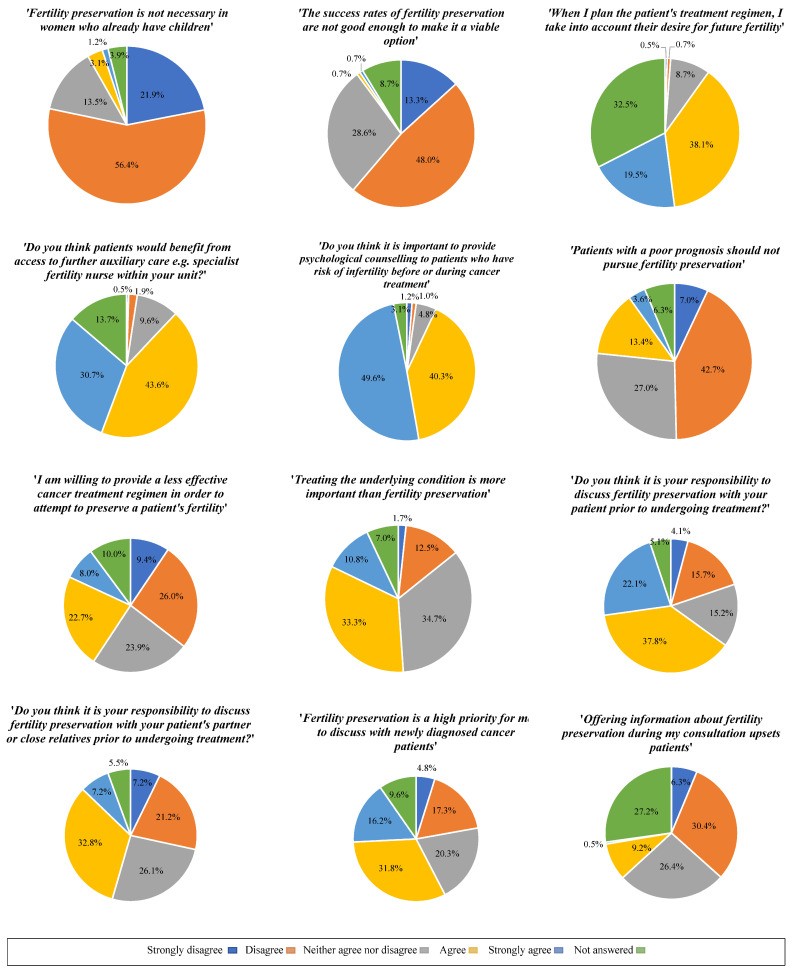
Attitudes towards fertility preservation techniques.

For the following age groups, participants reported this was the age up to which they would offer FP: 20–25 (5.3%; n = 22), 26–30 (6.9%; n = 29), 31–35 (12.5%; n = 52), 36–40 (45.3%; n = 188), 41–45 (22.7%; n = 94), 46–50 (6.3%; n = 26) and 1% (n = 4) did not answer. When asked if participants felt it was their responsibility to discuss FP with their patient prior to undergoing treatment, 4.1% (n = 17) answered strongly disagree, 15.7% (n = 65) disagree, 15.2% (n = 63) neither agreed nor disagreed, 37.8% (n = 157) agreed, 22.2% (n = 92) strongly agreed and 5.1% (n = 21) did not answer. When asked if participants felt it was their responsibility to discuss FP with their patient’s partner or close relatives prior to undergoing treatment, 7.2% (n = 30) answered strongly disagree, 21.2% (n = 88) disagree, 26.0% (n = 108) neither agreed nor disagreed, 32.8% (n = 136) agreed, 7.2% (n = 30) strongly agreed and 5.5% (n = 23) did not answer. When asked to rate the statement ‘fertility preservation is a high priority for me to discuss with the partner or relative of a newly diagnosed cancer patient’, 9.4% (n = 39) answered strongly disagree, 26.0% (n = 108) disagree, 23.9% (n = 99) neither agreed nor disagreed, 22.7% (n = 94) agreed, 7.9% (n = 33) strongly agreed and 10.1% (n = 42) did not answer. Figure 5 reports the cohort’s attitudes towards FP through various questions answered using a Likert scale. 

### 3.4. Barriers to Fertility Preservation

**Figure 6:**
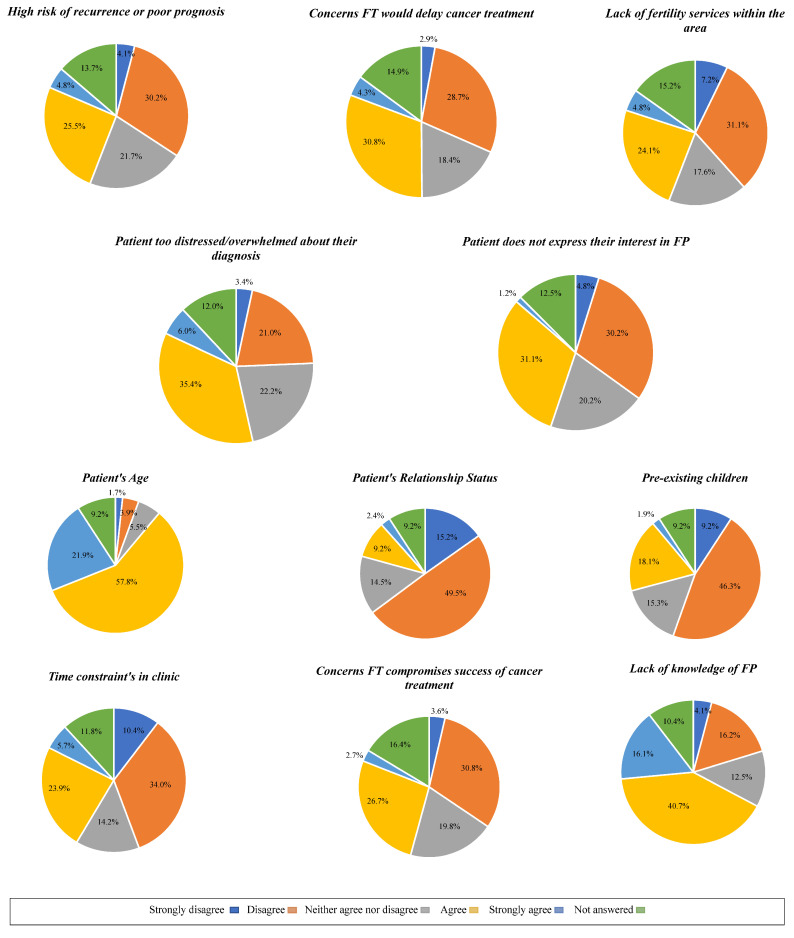
Barriers to discussing fertility perseveration as perceived by healthcare professionals.

Figure 6 reports the perceived barriers HCPs face when discussing FP with patients following a diagnosis of cancer. 

### 3.5. Knowledge of Local Fertility Preservation Services

**Figure 7:**
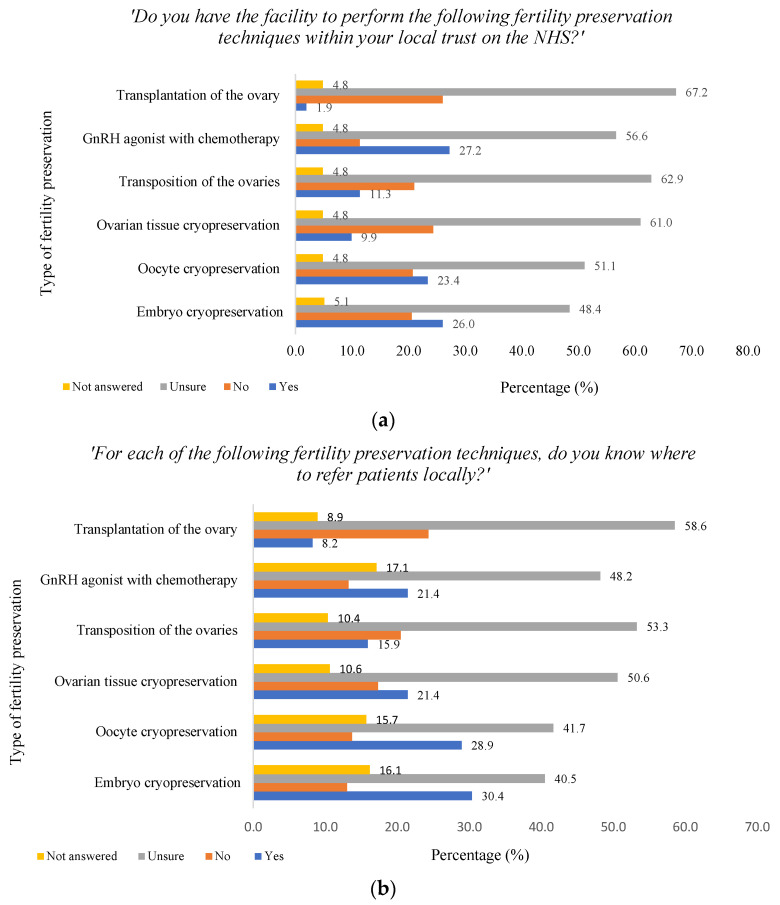
Knowledge of local fertility preservation services. (**a**) Knowledge of fertility preservation techniques within loacal trust. (**b**) Knowledge of referral to specialist unit.

Figure 7a reports whether participants had knowledge of certain FP techniques available in their local trust on the NHS for cancer patients and Figure 7b reports if participants knew where to refer patients for various FP techniques locally. In regard to whether there were clear referral pathways within the participants’ local trust to FP specialists, 33.3% (n = 138) reported yes, 12.8% (n = 53) reported no and the majority answered they were unsure 48.2% (n = 200), with 5.8% (n = 24) not answering. When asked if there were local guidelines on FP in women of reproductive age diagnosed with cancer, 16.4% (n = 68) reported yes, 13.9% (n = 58) reported no, 65.8% (n = 273) were unsure and 3.9% (n = 16) did not answer. Supplementary Figure S3 reports how participants rated access for patients to FP services within their unit and trust. When asked how they would describe their professional links with the local reproductive medicine unit, 3.4% (n = 14) answered very poor, 13.0% (n = 54) poor, 31.1% (n = 129) fair, 27.2% (n = 113) good, 8.4% (n = 35) excellent, 15.7% (n = 65) answered not applicable and 1.2% (n = 5) did not answer. 

### 3.6. Training on Fertility Preservation

**Figure 8:**
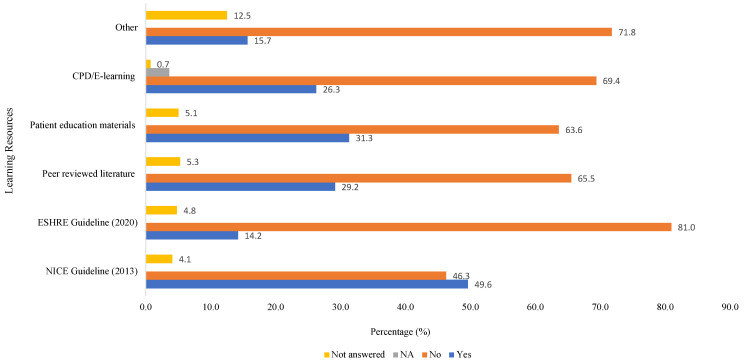
Learning resources participants have consulted with for further information on fertility preservation.

The majority (87.2%; n = 362) of participants had not experienced any formal training on FP compared to 9.4% (n = 39) who had, whereas 2.7% (n = 11) answered not applicable to the question and 0.7% (n = 3) did not answer. The majority (82.2%; n = 341) felt they personally needed more knowledge or formal training on FP options compared to 12% (n = 50), who felt this was not necessary, whereas 5.1% (n = 21) did not feel further knowledge and training was applicable to them and 0.7% (n = 3) did not answer. Overall the majority (74.2%; n = 308) felt their unit needed more knowledge or formal training on FP options compared to 12% (n = 50) who did not feel this was necessary, whereas 13% (n = 54) did not feel further knowledge and training was applicable to their unit and 0.7% (n = 3) did not answer. Figure 8 summarises the learning resources participants have consulted with in order to find further information about FP. 

## 4. Discussion

To our knowledge, this is the most recent survey carried out in the last 5 years assessing HCPs from various specialities on the awareness, attitudes and knowledge of FP in the UK. Our study highlights the majority of participants had limited experience of discussing the impact of a diagnosis of cancer on a patient’s future fertility. This is despite FP referrals increasing in the UK, with one study reporting an estimated 338–620 referrals in a 12-month period amongst all Principal Treatment Centres (PTCs) for children less than 18 years old [32]. Similarly, across Europe, data from the FertiPROTEKT registry, a multinational network from Germany, Austria and Switzerland with 85 registered centres, demonstrated that the number of individuals who have been counselled on FP has significantly increased over the years, from 388 in 2007 to 1043 in 2013 (*p* < 0.001) [33]. Our results may be attributed to the fact it is mainly senior doctors (ST6 and above), sub-specialist trainees or consultants who are likely to see patients diagnosed with cancer and expected to provide counselling. However, HCPs with this level of higher training accounted for only 38.1% of our cohort. In a previous study, the majority of consultant oncologists and Haematologists lacked knowledge regarding the duration of funding available for all FP options within their own centres [32]. It is unsurprising and perhaps overly ambitious, therefore, for junior clinicians without sub-specialist knowledge of FP or experience, to possess the skills and expertise to counsel patients adequately. Evidently, even at the highest level of training, where exposure to cancer patients requiring FP is frequent, further education is still required to ensure patients are offered optimal methods of treatment. 

Through a number of national and international networks and societies, such as the European Society of Human Reproduction and Embryology (ESHRE), the Fertility Consortium in the USA and the International Society of Fertility Preservation, emerging evidence has demonstrated that FP methods, previously deemed experimental, such as the transplantation of ovarian tissue and administration of GnRH agonists, are effective treatments for FP in cancer patients [34,35]. However, the techniques require expertise from both reproductive specialists and oncologists, and therefore multi-disciplinary and multicentre networks are paramount in the effective implementation of services. Hence, why within our own findings junior HCPs were unlikely to manage such patients. To offer individualised treatment, the ultimate goal would be that each oncology patient is discussed by a multi-disciplinary team [36] and their options of FP are considered case by case. The UK, however, currently lacks an effective method of integrating expertise from multiple centres or the ability to evaluate a national register of data. In the context of paediatric cancer, for example, services are currently delivered through 20 specialist PTCs, with no standardised NHS-commissioned or funded FP services for children in the UK. Furthermore, where NHS-funded treatment such as sperm collection and storage is offered, it is subject to the individual Clinical Commissioning Groups (CCGs), resulting in significant inequality and geographical variation in service provision across the country [32]. Evidently, there is an argument for streamlining services and centralising funding, as well as providing a national advisory panel to support PTCs [32], to improve implementation and equality of services. Although almost half of our cohort rated current access to FP services within their trust and, or unit, as adequate, various studies show that patient experiences are less positive [37,38]. Reasons for dissatisfaction include having to visit multiple medical facilities, engaging in numerous conversations with more than one HCP and feeling inundated by too much information [39]. Furthermore, very few HCPs in our study provided patients with written information, most likely because they lacked knowledge of appropriate educational resources to direct patients towards [40,41]. This is important considering patients often demand patient-specific written information about FP options available to them [38]. Should the centralisation of FP occur, patients would benefit from not only standardisation of information provided during counselling, but also a more holistic approach, so they feel less overwhelmed at the time of diagnosis, which may enhance patient experience [7,42].

There are also significant inequalities with respect to the type of FP offered across the country, with one study demonstrating that 100% of the PTCs had NHS funding to perform sperm banking, whereas only 60% of the centres had knowledge of funding pathways for procedures, such as oocyte cryopreservation [32]. Given that PTCs might not offer all methods of FP and are dependent on funding, often resulting in referrals to multiple centres, this could explain why almost half the HCPs in our study were unsure whether their local NHS trust offered each type of FP method, or did not know where to refer patients locally for treatment. This is consistent with other studies where HCPs lacked knowledge of how to refer and who the local fertility unit is [43], available facilities within the area [23,42,43,44,45,46] and knowledge of local guidelines [40]. Evidently, there is a demand for clearer referral pathways within areas, which could improve the efficiency of services significantly and delay the initiation of FP treatment.

Various studies have identified a lack of knowledge as a primary barrier to initiating discussions regarding FP [47,48,49], as reflected by our own study, where just over a third felt confident of the risks of infertility treatments. Gaps in knowledge often relate to the type of FP procedures available [40,50], which, despite development in ART, remains a current cause for concern, as exemplified by the majority of participants reporting their knowledge as ‘very poor’ or ‘poor’ for each method. This is particularly concerning because a lack of knowledge is associated with personal discomfort, which influences the likelihood of discussion of the topic [23]. It is important, therefore, that HCPs working with patients of reproductive age update their knowledge annually, possibly through continual professional development (CPD) courses recognised by their professional bodies to ensure they provide patients with accurate information, or can at least direct them to specialist FP services.

Further discrepancies in knowledge are related to the balance between oncological prognosis and perceived success of FP methods [51,52]. When HCPs perceive a poor oncological prognosis, they are more likely to deter discussions on fertility [51,52], which inadvertently challenges the recommended patient-centred approach to the treatment of cancer. Also, when HCPs believe oncological treatment is urgent, and in cases of aggressive or unusual cancer types, they are often uncertain how the duration of fertility treatment would impact cancer treatment [53]. Our results strongly suggest that HCPs have improved awareness of cancer survival rates and success from FP treatments, and perhaps no longer deem FP a secondary objective, as over half the cohort agreed they ‘consider patients reproductive aspirations when planning treatment of cancer.’ This is certainly an improvement from studies demonstrating that HCPs often overestimated the importance of cancer prognosis and survival, prioritising this over risks of infertility [46,54]. Furthermore, a study demonstrated that 48% of 718 oncology staff reported they had either never initiated discussion or offered sperm cryopreservation treatment to less than a quarter of eligible men because of their perceived poor prognosis [55]. Only a minority of respondents overall agreed that the risk of recurrence or poor prognosis was a barrier to FP, which suggests they are aware of national guidance that recommends all cancer patients of reproductive age should be counselled on FP treatment prior to undergoing cancer therapy, regardless of their prognosis.

Other prominent barriers to implementing FP in our study included perceived distress following a diagnosis and the patient’s age. This is consistent with a study where HCPs perceived that initiating discussions of FP, or the potential loss of fertility due to cancer treatment would increase the patient’s or family member’s anxiety when they were already in a state of shock from their diagnosis [23]. Such feelings lead to avoidance behaviour, where HCPs do not discuss important aspects of care during their consultation. However, given that a small proportion of the cohort agreed or strongly agreed that ‘offering information about FP upsets patients,’ suggests that HCPs may recognise this is a concern, but equally deem it a necessity to still engage in discussions regardless. A recent systematic review also highlighted factors such as age, relationship status and sociodemographic factors influence HCP discussions about FP, resulting in biased clinical practice [48]. However, in view of many individuals now delaying childbearing, increased rates of divorce, second marriages and the evolving modern family structure, it is important to challenge such misconceptions and beliefs amongst HCPs. The majority of HCPs reported they would offer FP to women aged between 36 and 50 and overall disagreed with the statement ‘FP is not necessary in women who already have children,’ suggesting that they are aware of the above misconceptions and mindful that many women delay childbearing. Regardless of the patient’s sociodemographic background, all patients of reproductive age are entitled to receive information and support about FP.

In regard to attitudes towards FP, our study shows that the majority of HCPs feel it is their responsibility to discuss the issue with their patients. This is a notable improvement to previous studies, where certain HCPs including oncology social workers, did not feel it was their initial responsibility [56,57]. Furthermore, in another study, surgeons felt that oncologists should take ownership of discussing the risks of treatment to fertility, as they had more clinical knowledge to do so [58]. Given very few HCPs refer patients for FP frequently, either due to a lack of knowledge of referral pathways or services available, they may perceive that they are the patient’s only opportunity to discuss FP prior to commencing treatment of cancer.

Overall training of FP amongst HCPs was deemed very poor. The majority of HCPs however, felt that formal training is necessary, in order to improve knowledge both personally and within their department. As previously highlighted, one of the primary barriers to implementing FP are concerns regarding patient anxiety when initiating discussions, with many HCPs demonstrating avoidance behaviour and refraining from giving information. In a study of 167 oncologists, clinicians were frequently noted to use euphemisms, or intentionally withhold information to avoid disclosing a poor prognosis in order to preserve hope for the patient [59]. Similarly, there is also a relationship between personal discomfort perceived by the clinician when breaking bad news (BBN) and FP discussion behaviour [23]. This is reflected by a study where those who had received formal training in managing pain felt more comfortable managing the condition compared to those without [60]. Although training should improve general knowledge of FP services, such as procedures available, how to refer and awareness of local resources, there should also be an emphasis on how to communicate difficult news to patients, as this skill alone would overcome many of the perceived barriers faced by HCPs. However, few training programmes or guidelines specifically guide HCPs on how to approach difficult discussions [61,62]. It has also been acknowledged that across neonatology and obstetric specialities, there are studies on the formal training of BBN in everyday clinical practice [63,64,65]. Other than undergraduate training in the UK however, where the teaching of BBN through the use of acronyms such as ‘SPIKES’ occurs, little time is dedicated to this at the postgraduate level [66]. Structured training within a curriculum can improve students’ performance of BBN and increase their confidence and empathy when doing so [64]. Furthermore, evidence from a randomised controlled trial suggests that a combination of theoretical training and simulation practice followed by 1:1 debriefing for BBN amongst trainees in obstetrics and gynaecology is an effective resource for teaching communication skills, as reflected by improved verbal and non-verbal skills and perceived self-confidence reported by the participants [65]. As such, royal colleges should implement structured training and revalidation of communication skills throughout the years of the programme to improve the skills required to initiate challenging discussions, such as FP in cancer patients.

### Limitations and Strengths

To our knowledge, this is the most recent study of HCPs awareness of female fertility preservation services, across multiple specialities within the UK. Limitations of this study include the online nature of participant recruitment, which may be subject to selection bias, as younger participants are more likely to use social media than older, thus limiting the generalisability of the findings. This is reflected by the fact that 55.9% of the cohort were <35 years of age. The majority of questions were also close-ended, which limits further exploration of ideas and attitudes. Further studies could include an in-depth exploration of the themes assessed through the process of interviews; however, data collection would be less efficient and more time-consuming.

## 5. Conclusions

Our study highlights the majority of HCPs who manage cancer patients of reproductive age and engage in discussions of FP are of a senior level given the specialist expertise required. Despite advanced training within their respective fields, discrepancies in knowledge remain regarding techniques of FP, referral pathways, local facilities offering services and existing educational resources, which prevent efficient implementation of services. The overall awareness of long-term survival rates following treatment of cancer has changed the perception amongst HCPs, with many recognising the importance of FP, despite the prognosis of cancer and their responsibility to initiate discussions. There is also improved knowledge that FP may not delay the treatment of cancer. Training of FP is scarce but could be overcome by regular teaching on communication skills such as BBN, which will improve feelings of personal discomfort HCPs experience when initiating challenging discussions on FP. Further studies should address how the centralisation of FP services within the UK and standardisation of care and resources across the country could potentially minimise many of the barriers experienced by HCPs.

## Figures and Tables

**Table 1:** Demographic characteristics.

	Number	(%)
**Age (Years)**		
20–25	3	0.7
26–30	82	19.8
31–35	147	35.4
36–40	88	21.2
41–45	26	6.3
46–50	28	6.7
51–55	18	4.3
>56	23	5.5
Not answered	0	0
**Ethnicity**		
White	267	64.3
Asian	72	17.3
Black	18	4.3
Mixed	20	4.8
Other	28	6.7
Would rather not say	8	1.9
Not answered	2	0.5
**Speciality**		
Obstetrics and Gynaecology (general)	196	47.2
Gynaecological Oncology (Sub-speciality trainee)	8	1.9
Reproductive Medicine (Sub-speciality trainee)	4	0.9
Urogynaecology (Sub-speciality trainee)	0	0
Maternal and Fetal medicine (Sub-speciality trainee)	5	1.2
General Practice	112	26.9
Clinical Oncologist	22	5.3
Medical Oncologist	26	6.3
Haematologist	6	1.4
Breast Surgeon	3	0.7
Paediatrician	18	4.3
Paediatric Oncologist	1	0.2
Gastroenterologist	4	0.9
General Surgeon	8	1.9
Not answered	2	0.5
**Current Position**		
Consultant	92	22.2
Speciality Registrar (ST3–ST5)	119	28.7
Speciality Registrar (ST6–ST7)	60	14.5
Speciality Senior House Officer (ST1–ST2)	48	11.6
Subspecialist trainee	6	1.4
Staff Grade Registrar	12	2.8
Staff Grade Senior House Officer	3	0.7
Associate Specialist	1	0.2
Other	67	16.1
Not answered	7	1.7
**Deanery working in**		
East Midlands	15	3.6
East of England	22	5.3
Kent Surrey and Sussex	28	6.7
London	63	15.2
North East and North Cumbria	31	7.5
North West	34	8.2
Oxford	4	0.9
Peninsula	12	2.9
Severn	11	2.7
West Midlands	27	6.5
Wessex	25	6.0
Yorkshire and Humber	33	7.9
Scotland (North)	28	6.7
Scotland (South East)	30	7.2
Scotland (East)	3	0.7
Scotland (West)	13	3.1
Wales	19	4.6
Northern Ireland	8	1.9
Not answered	9	20
**Qualifications**		
Membership of the Royal College of Physicians (MRCP)	29	6.9
Fellowship of the Royal College of Physicians (FRCP)	10	2.4
Membership of the Royal College of General Practice (MRCGP)	83	20
Membership of the Royal College of Obstetrics and Gynaecology (MRCOG)	114	27.5
Fellow of the Royal College of Obstetricians and Gynaecologists (FRCOG)	6	1.4
Membership of the Royal College of Surgeons (MRCS)	8	1.9
Fellow of the Royal College of Surgeons (FRCS)	3	0.7
Fellowship of the Royal College of Radiologists (FRCR)	19	4.6
Other	46	11.1
Not answered	97	23.4

## Data Availability

All data is available in the Section 3, figures, tables and Supplementary Material.

## Supplementary Materials

The following supporting information can be downloaded at: https://www.mdpi.com/article/10.3390/cancers16152649/s1, File S1: Questionnaire; Figure S1: Cancer pathology commonly seen; Figure S2: Healthcare professional satisfaction; Figure S3: How would you rate access to fertility preservation for patients within local unit and trust?
