# Supplementary material for: A Cross-Sectional Survey of Healthcare Professionals’ Knowledge, Attitude and Current Behaviours towards Female Fertility Preservation Services within the UK
Source: Cancers (Basel). 2024 Jul 25;16(15):2649. doi: 10.3390/cancers16152649 (PMC11311658; doi:10.3390/cancers16152649)
Supplement: Supplementary file 1 [file cancers-16-02649-s001.zip › cancers-3087071 supplementary figures.pdf]

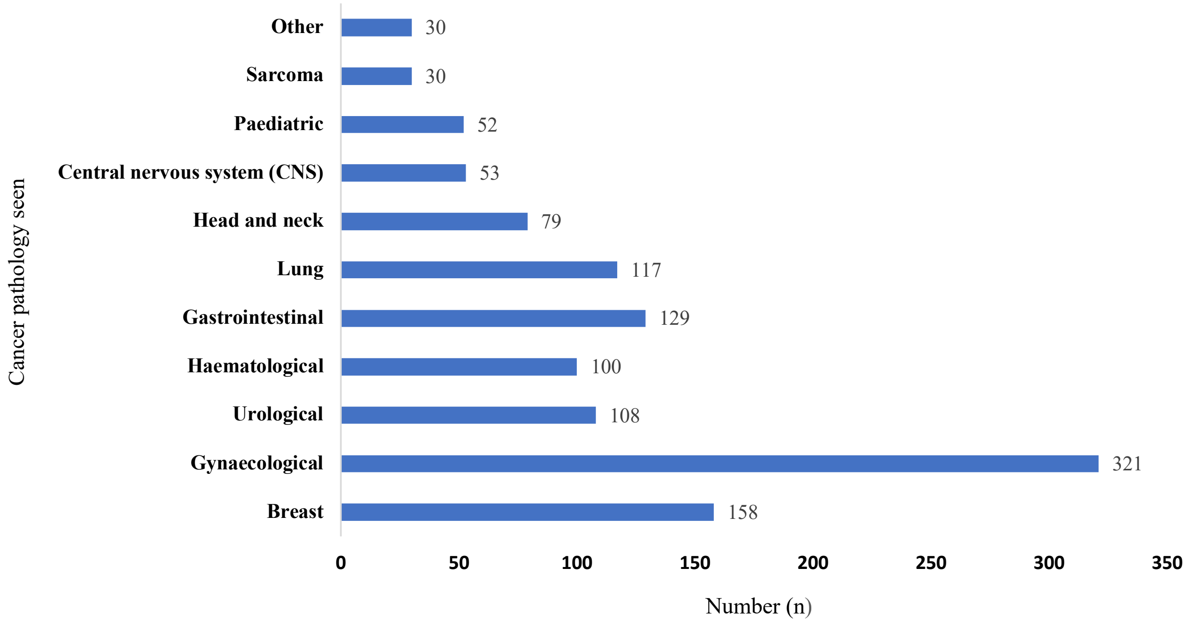

Figure S1. Cancer pathology commonly seen.

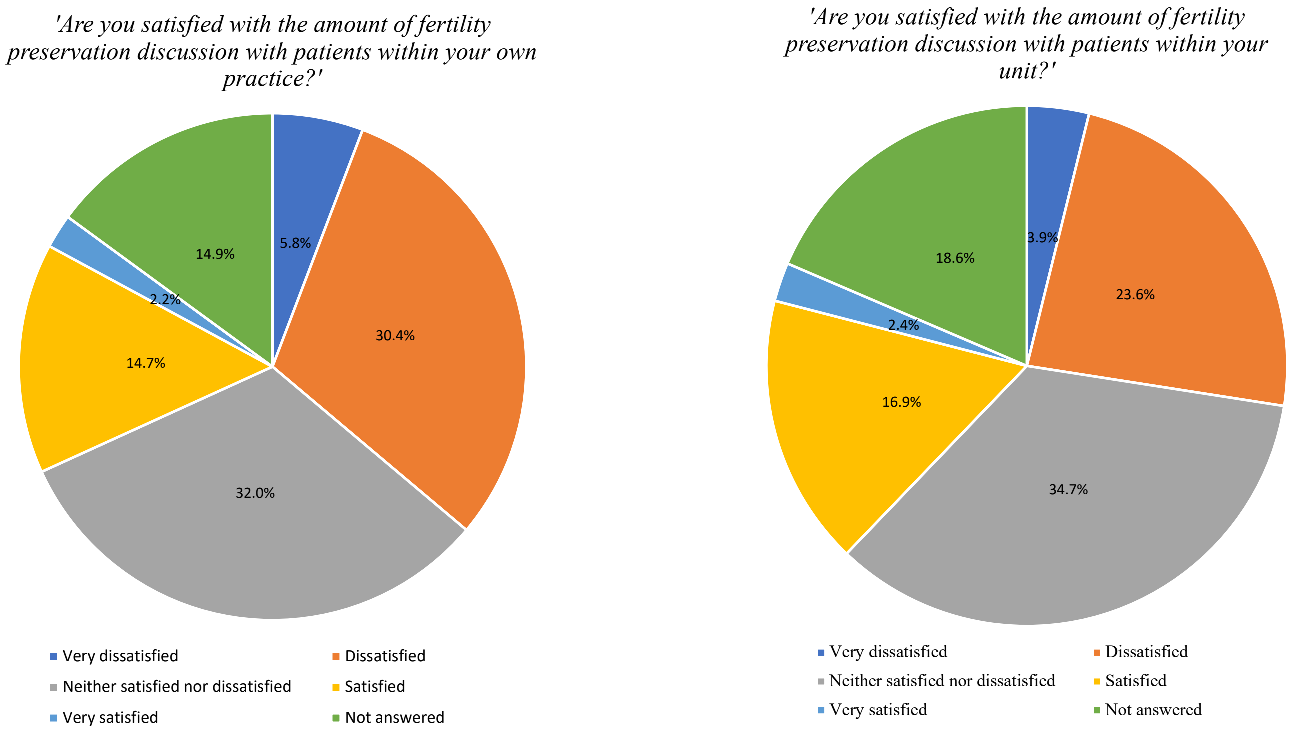

Figure S2. Healthcare professional satisfaction.

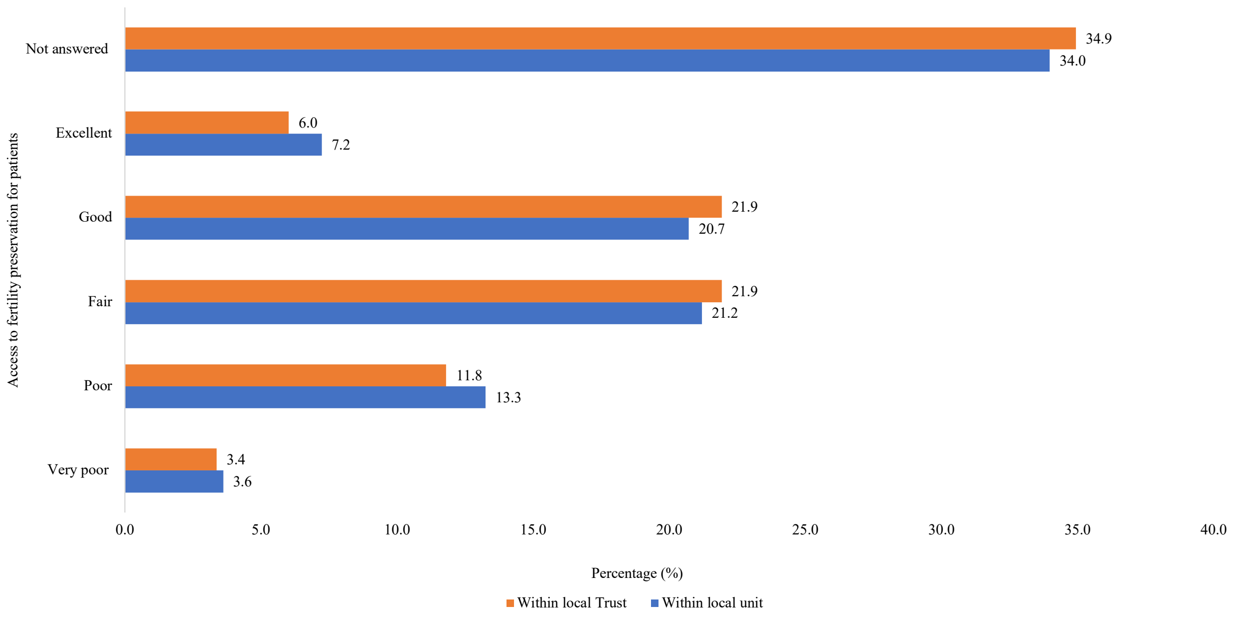

Figure S3. How would you rate access to fertility preservation for patients within local unit and trust?
