# Supplementary material for: A Cross-Sectional Survey of Healthcare Professionals’ Knowledge, Attitude and Current Behaviours towards Female Fertility Preservation Services within the UK
Source: Cancers (Basel). 2024 Jul 25;16(15):2649. doi: 10.3390/cancers16152649 (PMC11311658; doi:10.3390/cancers16152649)
Supplement: Supplementary file 1 [file cancers-16-02649-s001.zip › cancers-3087071-questionnaire.pdf]

Imperial College London

Supplementary materials

Questionnaire

(V2 – 2020)

A cross sectional survey of health care professionals' knowledge, attitude and current behaviours towards female fertility preservation services within the UK

1. Demographic information

a) Age (years)

- 20-25 ☐
- 26-30 ☐
- 31-35 ☐
- 36-40 ☐
- 41-45 ☐
- 46-50 ☐
- 51-55 ☐
- >56 ☐

b) Ethnicity

- White ☐
- Asian ☐
- Black ☐
- Mixed ☐
- Other ☐
- Would rather not say ☐

c) Speciality

Obstetrics and Gynaecology **(General)** ☐

Subspecialty if applicable:

- Gynaecological Oncology ☐
- Reproductive Medicine ☐
- Urogynaecology ☐
- Maternal and Fetal medicine ☐

General Practice ☐

Clinical Oncologist ☐
Medical Oncologist ☐
Haematologist ☐
Breast Surgeons ☐
Paediatrician ☐
Paediatric Oncologist ☐

d) **Current Position**

Consultant ☐
Speciality Registrar (ST3-ST5) ☐
Speciality Registrar (ST6-ST7) ☐
Speciality Senior House officer (ST1-ST2) ☐
Subspecialist Trainee ☐
Staff Grade registrar ☐
Staff Grade SHO ☐
Associate specialist ☐
Other ☐

e) **Did you complete your undergraduate training in the UK**

Yes ☐
No ☐

f) **Did you complete your speciality training in the UK?**

Yes ☐
No ☐

g) **Type of cancer pathology commonly seen (tick all)**

Breast ☐
Gynaecological ☐
Urological ☐
Haematological ☐
Gastrointestinal ☐
Lung ☐
Head and neck ☐
Central nervous system (CNS) ☐
Paediatric ☐
Sarcoma ☐
Other ☐

h) Deanery

East Midlands ☐
East of England ☐
Kent Surrey and Sussex ☐
London ☐
North East and North Cumbria ☐
North West ☐
Oxford ☐
Peninsula ☐
Severn ☐
West Midlands ☐
Wessex ☐
Yorkshire and Humber ☐

Scotland:
North ☐
South East ☐
East ☐
West ☐

Wales ☐
Northern Ireland ☐

i) Qualifications

MRCP ☐
FRCP ☐
MRGP ☐
MRCOG ☐
FRCOG ☐
MRCS ☐
FRCS ☐
FRCR ☐
Other ☐
Not applicable ☐

2. Current practice of fertility preservation

a) How often do you discuss the impact a patient's condition and or treatment might have on their future fertility?

Never ☐
Once a year ☐
Once a month ☐
Once a week ☐
Everyday ☐
Not applicable ☐

b) How often do you discuss the impact a patient's condition and or treatment might have on their future fertility with their partner/relatives/parents?

Never ☐
Once a year ☐
Once a month ☐
Once a week ☐
Everyday ☐
Not applicable ☐

c) How often do patients voluntarily initiate discussions regarding the risks of infertility related to treatment?

Never ☐
Once a year ☐
Once a month ☐
Once a week ☐
Everyday ☐
Not applicable ☐

d) How often do you discuss fertility preservation techniques when counselling patients prior to treatment?

Never ☐
Once a year ☐
Once a month ☐
Once a week ☐
Everyday ☐
Not applicable ☐

e) If so, what methods of fertility preservation techniques do you routinely discuss?

Embryo cryopreservation ☐
Oocyte cryopreservation ☐
Ovarian tissue cryopreservation ☐
Transposition of the ovaries ☐
GnRH agonist with chemotherapy ☐

Experimental techniques:
Transplantation of the ovary ☐

None of the above ☐
Not applicable ☐

f) How often do you refer patients to see a fertility specialist?

Never ☐
Once a year ☐
Once a month ☐
Once a week ☐
Everyday ☐
Not applicable ☐

g) How often do you consult a fertility specialist or a reproductive endocrinologist for advice with questions about potential fertility issues in your patients?

Never ☐
Once a year ☐
Once a month ☐
Once a week ☐
Everyday ☐
Not applicable ☐

h) Do you provide patients with written information about fertility preservation?

Yes ☐
No ☐
Not applicable ☐

i) Do you feel patents have enough access to written or online materials regarding fertility preservation e.g. leaflets?

Strongly disagree ☐
Disagree ☐

Neither agree nor disagree ☐
Agree ☐
Strongly agree ☐
Not applicable ☐

j) Do you feel that patients have enough access to fertility preservation auxiliary care e.g. specialist nurses?

Strongly disagree ☐
Disagree ☐
Neither agree nor disagree ☐
Agree ☐
Strongly agree ☐
Not applicable ☐

k) Are you satisfied with the amount of fertility preservation discussion with patients within your personal practice?

Very dissatisfied ☐
Dissatisfied ☐
Neither satisfied nor dissatisfied ☐
Satisfied ☐
Very satisfied ☐
Not applicable ☐

l) Are you satisfied with the amount of fertility preservation discussion with patients within your unit?

Very dissatisfied ☐
Dissatisfied ☐
Neither satisfied nor dissatisfied ☐
Satisfied ☐
Very satisfied ☐
Not applicable ☐

3. Knowledge of Fertility Preservation

a) Are you aware of the risks of infertility with the treatments you offer to patients of reproductive age routinely? (surgical or medical)

Not at all aware ☐
Slightly aware ☐
Moderately aware ☐
Very aware ☐
Extremely aware ☐
Not applicable ☐

b) How would you agree with the following statements:

'I feel confident to counsel a woman of reproductive age about fertility preservation?'

Strongly disagree ☐
Disagree ☐
Neither agree nor disagree ☐
Agree ☐
Strongly agree ☐
Not applicable ☐

c) How would you describe your level of knowledge of the following fertility preservation techniques:

Embryo cryopreservation:

Very poor ☐
Poor ☐
Fair ☐
Good ☐
Excellent ☐
Not applicable ☐

Oocyte cryopreservation:

Very poor ☐
Poor ☐
Fair ☐
Good ☐
Excellent ☐
Not applicable ☐

Ovarian tissue cryopreservation:

Very poor ☐
Poor ☐
Fair ☐
Good ☐
Excellent ☐
Not applicable ☐

Transposition of the ovaries:

Very poor ☐
Poor ☐
Fair ☐
Good ☐
Excellent ☐
Not applicable ☐

GnRH agonist with chemotherapy:

Very poor ☐
Poor ☐
Fair ☐
Good ☐
Excellent ☐
Not applicable ☐

Experimental techniques

Transplantation of the ovary:

Very poor ☐
Poor ☐
Fair ☐
Good ☐
Excellent ☐
Not applicable ☐

d) Do you think fertility preservation would delay the patient undergoing treatment for their underlying condition?

Yes ☐
No ☐
Unsure ☐
Not applicable ☐

e) **Are you aware of the health risks to the mother associated with pregnancy after completing treatment for cancer?**

- Yes ☐
- No ☐
- Unsure ☐
- Not applicable ☐

4. <u>Attitudes towards fertility preservation</u>

a) Do you think it is your responsibility to discuss fertility preservation with your patient prior to undergoing treatment?

- Strongly disagree ☐
- Disagree ☐
- Neither agree nor disagree ☐
- Agree ☐
- Strongly agree ☐
- Not applicable ☐

b) Do you think it is your responsibility to discuss fertility preservation with your patient's partner/close relatives prior to undergoing treatment?

- Strongly disagree ☐
- Disagree ☐
- Neither agree nor disagree ☐
- Agree ☐
- Strongly agree ☐
- Not applicable ☐

c) Which of the following best represents the age up to which you would consider fertility preservation advice relevant for a woman?

- 20-25 ☐
- 26-30 ☐
- 31-35 ☐
- 36-40 ☐
- 41-45 ☐
- 46-50 ☐

d) How would you rate the following statements:

'Fertility preservation is a high priority for me to discuss with newly diagnosed cancer patients'

Strongly disagree ☐
Disagree ☐
Neither agree nor disagree ☐
Agree ☐
Strongly agree ☐
Not applicable ☐

e) **'Fertility preservation is a high priority for me to discuss with the partner or relative of a newly diagnosed cancer patient'**

Strongly disagree ☐
Disagree ☐
Neither agree nor disagree ☐
Agree ☐
Strongly agree ☐
Not applicable ☐

f) **'I am willing to provide a less effective cancer treatment regimen in order to attempt to preserve a patient's fertility'**

Strongly disagree ☐
Disagree ☐
Neither agree nor disagree ☐
Agree ☐
Strongly agree ☐
Not applicable ☐

g) **'Treating the underlying condition is more important than fertility preservation'**

Strongly disagree ☐
Disagree ☐
Neither agree nor disagree ☐
Agree ☐
Strongly agree ☐
Not applicable ☐

h) **'When I plan the patient's treatment regimen I take into account their desire for future fertility'**

Strongly disagree ☐
Disagree ☐
Neither agree nor disagree ☐

Agree ☐
Strongly agree ☐
Not applicable ☐

i) 'Patients with a poor prognosis should not pursue fertility preservation'

Strongly disagree ☐
Disagree ☐
Neither agree nor disagree ☐
Agree ☐
Strongly agree ☐
Not applicable ☐

j) 'Offering information about fertility preservation during my consultation upsets patients'

Strongly disagree ☐
Disagree ☐
Neither agree nor disagree ☐
Agree ☐
Strongly agree ☐
Not applicable ☐

k) 'Fertility preservation is not necessary in women who already have children'

Strongly disagree ☐
Disagree ☐
Neither agree nor disagree ☐
Agree ☐
Strongly agree ☐
Not applicable ☐

l) 'The success rates of fertility preservation are not good enough to make it a viable option'

Strongly disagree ☐
Disagree ☐
Neither agree nor disagree ☐
Agree ☐
Strongly agree ☐

m) Do you think it is important to provide psychological counselling to patients who have risk of infertility before or during cancer treatment?

Strongly disagree ☐
Disagree ☐

Neither agree nor disagree ☐
Agree ☐
Strongly agree ☐

n) Do you think patients would benefit from access to further auxiliary care e.g. specialist fertility nurse within your unit?

Strongly disagree ☐
Disagree ☐
Neither agree nor disagree ☐
Agree ☐
Strongly agree ☐
Not applicable ☐

5. <u>Barriers to fertility preservation</u>

a) **What factors influence your decision to discuss fertility preservation:**

Patient's age

Strongly disagree ☐
Disagree ☐
Neither agree nor disagree ☐
Agree ☐
Strongly agree ☐
Not applicable ☐

b) **Relationship status of the patient**

Strongly disagree ☐
Disagree ☐
Neither agree nor disagree ☐
Agree ☐
Strongly agree ☐
Not applicable ☐

c) **If the patient already has children**

Strongly disagree ☐
Disagree ☐
Neither agree nor disagree ☐

Agree ☐
Strongly agree ☐
Not applicable ☐

d) **Time constraints in clinic**

Strongly disagree ☐
Disagree ☐
Neither agree nor disagree ☐
Agree ☐
Strongly agree ☐
Not applicable ☐

e) **Lack of knowledge regarding available options**

Strongly disagree ☐
Disagree ☐
Neither agree nor disagree ☐
Agree ☐
Strongly agree ☐
Not applicable ☐

f) **Concerns that fertility treatment would compromise success of cancer treatment**

Strongly disagree ☐
Disagree ☐
Neither agree nor disagree ☐
Agree ☐
Strongly agree ☐
Not applicable ☐

g) **Concerns that fertility treatment would delay cancer treatment**

Strongly disagree ☐
Disagree ☐
Neither agree nor disagree ☐
Agree ☐
Strongly agree ☐

Not applicable ☐

h) **Lack of fertility services within the area**

Strongly disagree ☐
Disagree ☐
Neither agree nor disagree ☐
Agree ☐
Strongly agree ☐
Not applicable ☐

i) **Patient does not express their interest**

Strongly disagree ☐
Disagree ☐
Neither agree nor disagree ☐
Agree ☐
Strongly agree ☐
Not applicable ☐

j) **The patient has a high risk of recurrence or poor prognosis**

Strongly disagree ☐
Disagree ☐
Neither agree nor disagree ☐
Agree ☐
Strongly agree ☐
Not applicable ☐

k) **The patient appears distressed or overwhelmed about her diagnosis and or treatment**

Strongly disagree ☐
Disagree ☐
Neither agree nor disagree ☐
Agree ☐
Strongly agree ☐
Not applicable ☐

6. Knowledge of local fertility preservation services

a) Do you have the facility to perform the following fertility preservation techniques within your local trust on the NHS?

Embryo cryopreservation

Yes ☐
No ☐
Unsure ☐
Not applicable ☐

Oocyte cryopreservation

Yes ☐
No ☐
Unsure ☐
Not applicable ☐

Ovarian tissue cryopreservation

Yes ☐
No ☐
Unsure ☐
Not applicable ☐

Transposition of the ovaries

Yes ☐
No ☐
Unsure ☐
Not applicable ☐

GnRH agonist with chemotherapy

Yes ☐
No ☐
Unsure ☐
Not applicable ☐

Experimental techniques:
Transplantation of the ovary

Yes ☐
No ☐
Unsure ☐

Not applicable ☐

b) **If not, for each of the following fertility preservation techniques, do you know where to refer patients locally?**

Embryo cryopreservation

Yes ☐
No ☐
Unsure ☐
Not applicable ☐

Oocyte cryopreservation

Yes ☐
No ☐
Unsure ☐
Not applicable ☐

Ovarian tissue cryopreservation

Yes ☐
No ☐
Unsure ☐
Not applicable ☐

Transposition of the ovaries

Yes ☐
No ☐
Unsure ☐
Not applicable ☐

GnRH agonist with chemotherapy

Yes ☐
No ☐
Unsure ☐
Not applicable ☐

Experimental techniques-Transplantation of the ovary

Yes ☐
No ☐
Unsure ☐

Not applicable ☐

c) **Do you have clear referral pathways within your trust to fertility preservation specialists?**

Yes ☐
No ☐
Unsure ☐
Not applicable ☐

d) **Does your trust have any local guidelines regarding fertility preservation in women of reproductive age diagnosed with cancer?**

Yes ☐
No ☐
Unsure ☐
Not applicable ☐

e) **How would you rate access to fertility preservation for patients within your unit?**

Very poor ☐
Poor ☐
Fair ☐
Good ☐
Excellent ☐
Not applicable ☐

f) **How would you rate access to fertility preservation for patients within your local trust?**

Very poor ☐
Poor ☐
Fair ☐
Good ☐
Excellent ☐
Not applicable ☐

g) **Which of the following best describes your professional links with the reproductive medicine unit that is closest to you?**

Very poor ☐
Poor ☐
Fair ☐
Good ☐
Excellent ☐

Not applicable ☐

7. **Training of Fertility preservation**

a) **Have you ever had formal training on fertility preservation?**

Yes ☐
No ☐
Not applicable ☐

b) **Do you feel you need more knowledge/formal training on fertility preservation options?**

Yes ☐
No ☐
Not applicable ☐

c) **Do you feel your unit needs more knowledge/formal training on fertility preservation options?**

Yes ☐
No ☐
Not applicable ☐

d) **Have you consulted any of the following resources for further information on fertility issues?**

NICE fertility guidelines CG156 (2013)

Yes ☐
No ☐
Not applicable ☐

ESHRE Female Fertility Preservation Guideline 2020

Yes ☐
No ☐
Not applicable ☐

Peer reviewed literature

Yes ☐
No ☐
Not applicable ☐

Patient education materials

Yes ☐
No ☐
Not applicable ☐

Education materials provided by professional training body (e.g. CPD/E- learning modules)

Yes ☐
No ☐
Not applicable ☐

Other

Yes ☐
No ☐
Not applicable ☐
